# Preliminary Investigation into a Novel Sustained-Release Formulation of Meloxicam in Sheep (*Ovis aries*)—Pharmacokinetic Profile

**DOI:** 10.3390/ani11092484

**Published:** 2021-08-24

**Authors:** Christine Plummer, Peter J. White, Benjamin Kimble, Merran Govendir, Dominique Van der Saag

**Affiliations:** 1 Faculty of Science, School of Life and Environmental Sciences, University of Sydney, Sydney, NSW 2006, Australia; cplu6736@uni.sydney.edu.au; 2 Faculty of Science, Sydney School of Veterinary Science, University of Sydney, Sydney, NSW 2006, Australia; benjamin.kimble@sydney.edu.au (B.K.); merran.govendir@sydney.edu.au (M.G.); dominique.van.der.saag@sydney.edu.au (D.V.d.S.)

**Keywords:** meloxicam, sheep, pharmacokinetics

## Abstract

**Simple Summary:**

Meloxicam is an effective non-steroidal anti-inflammatory drug (NSAID) suitable for ameliorating pain in sheep. Pain caused by husbandry procedures and other inflammatory conditions in sheep can persist for an extended time beyond the duration of action of currently available formulations of NSAIDs. This study investigates a novel sustained-release formulation of meloxicam to determine its potential for extended pain alleviation. Compared to a conventional formulation of meloxicam, the sustained-release formulation provided extended half-life making it a suitable candidate for providing extended pain relief.

**Abstract:**

This study is a preliminary investigation describing the pharmacokinetic profile of a novel subcutaneous sustained-release meloxicam formulation (SRMF) in sheep. Six merino ewe hoggets (41.5 ± 4.6 kg) were treated with a novel subcutaneous SRMF at 2 mg/kg bodyweight (BW). Blood samples were collected at t = 0, 2, 4, 6, 8, 10, 12, 24, 48, 96, 144, 168, 192, and 336 h following treatment, and interstitial (ISF) fluid samples were collected at periods of 8 to 12 h, 12 to 24 h, 24 to 48 h, 48 to 52 h, and 92 to 96 h following treatment. High-pressure liquid chromatography (HPLC) analysis with ultraviolet detection was utilised to determine the concentration of meloxicam in plasma and ISF. The SRMF exhibited the following mean (±SD) pharmacokinetic indices: C_max_ of 1.58 μg/mL (±0.82 μg/mL) at a T_max_ of 10.0 h (±1.79 h), and half life (t_1/2_) of 31.4 h (±13.17 h) in sheep plasma. Interstitial fluid samples were collected from three of the six sheep, with a decrease in meloxicam concentration exhibited over 52 h. This study demonstrates a variable extended t_1/2_, a delayed T_max_, and a lower C_max_ of the SRMF, as compared to that of a conventional meloxicam formulation (CMF) in sheep, as previously referenced (t_1/2_: 14.28 h; T_max_: 5 h; C_max_: 15.94 μg/mL). Further research to determine the clinical efficacy and safety of the SRMF in sheep is warranted.

## 1. Introduction

Meloxicam is an enolic acid that belongs to the oxicam class of non-steroidal anti-inflammatory drugs (NSAIDs) [1]. Meloxicam possesses a higher therapeutic index than other NSAIDs due to its analgesic, anti-inflammatory, and antipyretic properties [2,3]. Through inflammatory provocation, meloxicam preferentially suppresses the cyclo-oxygenase 2 (COX-2) pathway, inhibiting the conversion of arachidonic acid into prostaglandins [1,2]. Meloxicam possesses various beneficial actions that make it a model NSAID for use in animals, including extended elimination half-life, low gastrointestinal toxicity, suitable oral and injectable absorption rates, and good bioavailability [2,3,4]. The pharmacokinetic (PK) profile of meloxicam has been studied in several companion, wildlife, and production species including koalas [5], guinea pigs [6], dogs [7], cats [8], birds [9], cattle [10], horses [11], and sheep [12,13]. 

The elimination half-life for a conventional meloxicam formulation (CMF), Metacam 20 (Boehringer Ingelheim, Ingelheim am Rhein, Germany), in sheep has been reported as mean (±SD) 14.28 ± 2.41 h when administered subcutaneously, and 12.79 ± 3.49 h when administered intramuscularly at 2 mg/kg [12]. Pain has been detected for several days following husbandry procedures in sheep, as indicated by introverted behaviours and elevated physiological markers of inflammation [14,15]. Thus, the duration of action of currently available formulations of meloxicam to address the pain of such procedures is inadequate. Extending the duration of action of meloxicam could address longer-lasting inflammatory pain without the requirement for repeated dosing, which is impractical on-farm and can cause further stress and potential damage to existing wounds in sheep. 

The objective of this study was to examine the pharmacokinetic profile of a novel sustained-release meloxicam formulation (SRMF) in sheep. The results of this study will determine whether the SRMF has a longer half-life in sheep compared to a conventional meloxicam formulation (CMF), as determined in a previous study [12].

## 2. Materials and Methods

### 2.1. Animals and Housing

The experiment was approved by The University of Sydney Animal Care and Ethics Committee (Approval number 2017/1215). The study utilised six Merino ewe hoggets sourced from The University of Sydney’s property, Mayfarm, in Camden, New South Wales, Australia. The sheep had a mean (±SD) weight of 41.5 kg (±4.6 kg). During the experimental period, sheep were housed in a sheltered yard (20 m × 10 m) with dirt flooring and straw bedding. All sheep were provided ad libitum access to lucerne hay and water. Sheep were returned to Mayfarm at the conclusion of the study. 

### 2.2. Treatment

All sheep were injected with a novel SRMF (60 mg/mL) (Australian Custom Pharmaceuticals Pty Ltd., Sydney, NSW, Australia) at a dose rate of 2 mg/kg bodyweight. The novel formulation, administered via a single subcutaneous (SC) injection using an 18 g needle, had been specifically formulated for the sustained release of meloxicam. The SRMF consisted of a biodegradable polymer and 60 mg/mL of meloxicam in a water-miscible organic solvent. This formulation forms an in situ solid bolus after subcutaneous injection, releasing meloxicam slowly from the polymer. Although it is common practice for SC injections to be administered into the neck of sheep, in this study, the SRMF was injected subcutaneously under the left forelimb. This was performed to allow physical distancing between the ultrafiltration sampling probes positioned in the dorsal neck and the injection site. 

### 2.3. Sample Collection

Blood samples (10 mL) were collected into lithium heparin vacutainers via jugular venipuncture using an 18 g needle. Blood samples were collected immediately prior to treatment (0 h), then at 2, 4, 6, 8, 10, 12, 24, 48, 96, 144, 168, 192, and 336 h following treatment. Following blood collection at each time point, samples were centrifuged at 1700× *g* for 7 min. Plasma was extracted and stored at −20°C until analysed.

Interstitial fluid (ISF) samples were collected from sheep using in vivo ultrafiltration sampling probes (RUF-3-12 Reinforced In Vivo Ultrafiltration Sampling Probe, BASI Research Products, Lafayette, IN, USA) implanted subcutaneously in the neck by insertion using a 14 g canula as a guide and then suturing in place. Vacutainers, attached to the probes for sample collection, were housed in a pouch on a collar placed around the sheep’s neck. Sampling probes were inserted immediately prior to treatment. Interstitial fluid was collected from vacutainers at 8 to 12 h, 12 to 24 h, 24 to 48 h, 48 to 52 h, and 92 to 96 h and was stored at −20°C until analysis. The maximum ISF collected at each interval was approximately 1 mL per sheep. These time periods were utilised to allow adequate time between sampling for collection of a sufficient volume of fluid for analysis.

### 2.4. Plasma and ISF Meloxicam Analysis 

High-pressure liquid chromatography (HPLC) analysis with ultraviolet detection was utilised to determine the concentration of meloxicam in the plasma and ISF samples as previously described [12].

### 2.5. Pharmacokinetic Analysis 

The PK profile was established through a noncompartmental model using PK Solver [16]. The indices of maximum observed plasma concentration (C_max_) and time taken to reach maximum plasma concentration (T_max_) were determined through visual comparison of the plasma concentration and time curve. The elimination rate constant (k_el_) was established through a semi-log regression of the terminal slope. The terminal half-life (t_1/2_) was determined as ln 2/k_el_. 

The area under the concentration-time curve (AUC0-t last) was calculated to the last measurable concentration (t_last_) using the trapezoidal method. The AUC and AUMC from the last observed concentration to infinity were determined by:

AUC_t–∞_ = C_last_/k_el_ AUMC_t–∞_ = (C_last_ × t_last_/k_el_) + (C_last_/k_el_^2^)

The mean residence time (MRT) was determined by:

MRT = AUMC_0–∞_/AUC_0–∞_

The apparent volume of distribution was determined by:

V/F = (Dose × AUMC)/AUC^2^

The apparent clearance was determined using:

CL/F = Dose/AUC

The amount of unbound meloxicam in the ISF was also quantified.

## 3. Results

**Figure 1:**
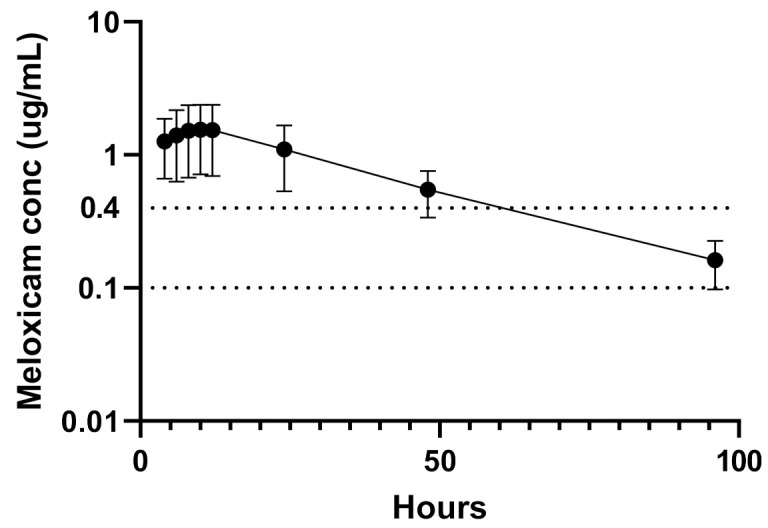
Plasma concentrations (ug/mL) of 2 mg/kg sustained-release meloxicam formulation (SRMF) following subcutaneous administration in sheep (*n* = 6) over 96 h. The dotted line at y = 0.1 μg/mL is the lower limit of quantification of the assay [12]. The dotted line at y = 0.4 μg/mL is a theoretical plasma analgesic concentration.

**Figure 2:**
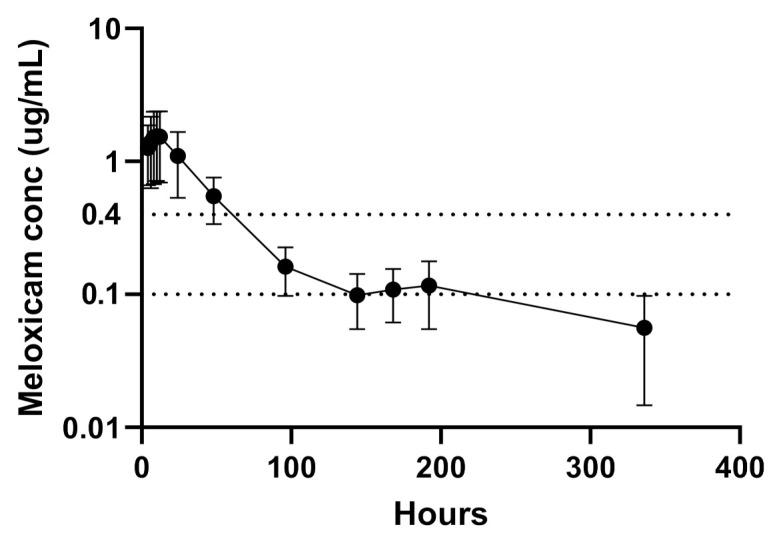
Plasma concentrations (ug/mL) of 2 mg/kg sustained-release meloxicam formulation (SRMF) following subcutaneous administration in sheep (*n* = 6) over time. The dotted line at y = 0.1 μg/mL is the lower limit of quantification of the assay [12]. The dotted line at y = 0.4 μg/mL is a theoretical plasma analgesic concentration.

The plasma PK indices are presented in Table 1. Plasma SRMF concentrations detected in each sheep over 96 h and over time are presented in Figure 1 and Figure 2, respectively.

**Figure 3:**
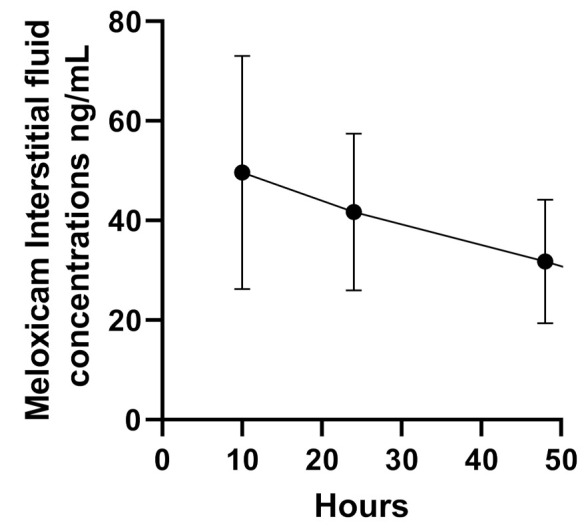
Interstitial fluid concentrations (ng/mL) of 2 mg/kg sustained-release meloxicam formulation (SRMF) following subcutaneous administration in sheep (*n* = 4).

The ISF concentrations of meloxicam are presented in Table 2. The ISF samples from sheep 1 and 6 were not collected and only one fluid sample was collected from sheep 2 at 48 to 52 h due to failure of the ultrafiltration probe. Fluid samples were successfully collected from sheep 3, 4, and 5, which showed a decrease in meloxicam concentration in the fluid samples over time. Meloxicam was not detectable in ISF from sheep 4 and 5 at 24 to 48 h and 48 to 52 h, respectively. The ISF meloxicam concentrations of sheep 2, 3, 4, and 5 are presented in Figure 3.

## 4. Discussion

The aim of a SRMF for use in sheep is to provide effective pain relief and a reduction in inflammation for an extended period when compared to a CMF. This offers a reduction in labour involved with repeated dosing for prolonged relief and benefits the welfare of animals. This study analysed the PK profile of a novel SRMF and demonstrated the longest t_1/2_ found for meloxicam in sheep to date, with detectable concentrations present in sheep plasma until the conclusion of the study (336 h). 

The plasma concentrations of the SRMF used in this study provided an extended t_1/2_ of 31.40 h comparative to the CMF t_1/2_ of 14.28 h, when both were administered at 2 mg/kg via SC injection [12]. In a recent study in sheep, it was found that the t_1/2_ for a SC injection of SRMF (10 mg/mL, Zoopharm, Fort Collins, CO, USA) administered at 1.5 mg/kg was 15.2 h [17]. This difference may be due to a difference in dose rate and/or due to differences in the formulation and/or individual animal differences. 

The T_max_ of the SRMF in this study (10 h) was twice that of the CMF reported by a previous study (5 h), demonstrating that the rate of absorption of the SRMF in this study was slower [12]. Similarly, another study using a SRMF was found to have a T_max_ of 6.70 h in sheep, which is longer than that for CMF but less than the T_max_ in this study [17]. 

The C_max_ of the SRMF used in this study was 10 times lower (1.58 μg/mL) than that for the CMF (15.94 μg/mL) established in a previous study using CMF [12]. This lowered C_max_ in the SRMF is indicative of the active component of the injection being slowly released. 

Previous studies have postulated the minimum therapeutic level of SRMF in sheep to be 0.4 μg/mL [17]. This concentration was based on a review of CMF efficacy research in multiple species as well as consultation with drug manufacturers [17]. In this current study, the SRMF provided 60 h where plasma levels were above 0.4 μg/mL. Efficacy studies are required to confirm the long-term efficacy of this SRMF. 

Variant factors between trial sheep were maintained to a minimum; age, health condition, breed, and sex were kept the same. Despite this, individual animal variances in meloxicam concentrations were demonstrated in the blood plasma and ISF analyses. Consistently, interindividual variance was found in studies of SRMF in Hispaniolan Amazon parrots and mice [18]. This variability was associated with differences in SRMF absorption rate in individuals, related with the site of injection and the formulation dispersion [18]. Variation in this study was most clearly seen in t_1/2_ and C_max_ measurements in the plasma. Undertaking this study as a cross-over design, using CMF as the control, may have assisted in the explanation of this variation and should be considered in further studies. Anecdotal observations of the SRMF bolus positioned in the SC tissue throughout the experiment showed a difference in circumference and height of the bolus across individuals, likely due to a varied rate of interindividual drug decay. Another contributing factor to the size of the bolus and potentially rate of decay was the placement of the SRMF bolus. Due to the placement of the ultrafiltration probes in the neck of sheep, the SRMF was injected under the left forelimb of the sheep to avoid false concentration readings. However, this placement may have contributed to the concentration inconsistencies due to locomotion and pressure placed on this site.

To the best of our knowledge, this is the first study to utilise ultrafiltration probes to sample ISF following meloxicam administration in sheep. Non-steroidal anti-inflammatories drugs are known for their generally low volume of distribution and high level of binding to plasma proteins, potentially slowing their elimination [19]. Consequently, this reduces drug tissue permeability [19]. Plasma protein binding concentrations are crucial for determining PK parameters and indices [20]. It is also recognised that NSAIDs can persist longer in exudate than in transudates and plasma [21]. However, plasma is utilised primarily due to the complexity associated with collecting unbound drug concentrations in tissues [20]. These unbound concentrations have greater pharmacological activity and relevance due to their diffusion to the site of action. While there needs to be improvements with the collection method used in this study, meloxicam could be detected in the ISF fluid in two of the sheep over the 24–48 h duration.

Several complications were encountered in the probe implantation protocol including probe detachment, incorrect implantation, and probe failure. Consequently, this resulted in varied fluid volume and sample numbers across individuals. However, this is not an uncommon occurrence when utilising ultrafiltration probes for ISF collection as ultrafiltration probes produced varied fluid volume in individual cattle due to probe occlusion, failure, and inconsistent positioning [22,23]. Future studies with improved probe retention and a larger sample size may reduce these interindividual differences. 

This study administered the SRMF at a dosage rate of 2 mg/kg BW. No adverse gross effects of this novel SRMF, such as gastrointestinal or renal damage [21], were identified in trial sheep. Further research in this field is required to quantify a safe and effective dose for SRMF in sheep. Research into alternate dosage rates will determine whether the current dose of SRMF should be adjusted to be effectual for extended analgesia. 

## 5. Conclusions

The novel SRMF examined in this study demonstrates a mean t_1/2_ of 31.4 h in sheep when administered subcutaneously under the forelimb. This formulation was detectable in plasma until the conclusion of the study at 336 h. The t_1/2_ of this formulation is the longest reported for meloxicam in sheep to date. Further investigation into the therapeutic dosage, efficacy, and safety of this SRMF is required to ascertain its potential use following routine husbandry procedures in sheep.

## Figures and Tables

**Table 1:** Plasma pharmacokinetic indices following subcutaneous administration of 2 mg/kg sustained-release meloxicam formulation (SRMF) in sheep (*n* = 6).

PK Indices	Sheep 1	Sheep 2	Sheep 3	Sheep 4	Sheep 5	Sheep 6	Mean	SD
T_max_ (h)	8.00	10.00	12.00	8.00	12.00	10.00	10	1.79
C_max_ (μg/mL)	2.80	2.41	1.4	1.06	0.85	0.95	1.58	0.82
AUC_0-t_ (μg/mL × h)	97.01	105.71	74.4	40.02	38.39	64.21	69.96	28.13
AUC_0-inf_ (μg/mL × h)	99.74	109.89	82.3	43.7	44.78	76.74	76.19	27.46
AUC_0-t_/AUC_0–∞_ %	97%	96%	90%	92%	86%	84%	91%	97%
AUMC_0-inf_ (μg/mL × h^2^)	2764.99	3520.26	4764.42	1660.06	2173.35	5930.36	3468.91	1623.93
MRT (h)	27.72	32.04	57.89	37.98	48.53	77.28	40.83	12.32
V/F (L/kg)	0.52	0.51	1.39	1.69	2.15	1.97	1.25	0.73
Cl/F (L/kg/h)	0.02	0.02	0.02	0.05	0.05	0.03	0.03	0.01

**Table 2:** Interstitial fluid meloxicam concentrations (ng/mL) following subcutaneous administration of 2 mg/kg sustained-release meloxicam formulation in sheep (*n* = 6).

Time Period (h)	Sheep 1	Sheep 2	Sheep 3	Sheep 4	Sheep 5	Sheep 6
8–12	No Sample	No Sample	42.27	30.8	75.8	No Sample
12–24	No Sample	No Sample	31.8	33.5	50.9	No Sample
24–48	No Sample	No Sample	27.2	No Peak Detected	46.2	No Sample
48–52	No Sample	40.52	23	No Peak Detected	No Peak Detected	No Sample
92–96	No Sample	No Sample	9	No Peak Detected	No Peak Detected	No Sample

## Data Availability

Not applicable.
